# Genetic basis of cytokinin and auxin functions during root nodule development

**DOI:** 10.3389/fpls.2013.00042

**Published:** 2013-03-11

**Authors:** Takuya Suzaki, Momoyo Ito, Masayoshi Kawaguchi

**Affiliations:** ^1^Division of Symbiotic Systems, National Institute for Basic Biology OkazakiAichi, Japan; ^2^Department of Basic Biology, School of Life Science, Graduate University for Advanced Studies (SOKENDAI), OkazakiAichi, Japan

**Keywords:** auxin, cytokinin, legume, nodulation, root nodule symbiosis

## Abstract

The phytohormones cytokinin and auxin are essential for the control of diverse aspects of cell proliferation and differentiation processes in plants. Although both phytohormones have been suggested to play key roles in the regulation of root nodule development, only recently, significant progress has been made in the elucidation of the molecular genetic basis of cytokinin action in the model leguminous species, *Lotus japonicus* and *Medicago truncatula*. Identification and functional analyses of the putative cytokinin receptors LOTUS HISTIDINE KINASE 1 and *M. truncatula* CYTOKININ RESPONSE 1 have brought a greater understanding of how activation of cytokinin signaling is crucial to the initiation of nodule primordia. Recent studies have also started to shed light on the roles of auxin in the regulation of nodule development. Here, we review the history and recent progress of research into the roles of cytokinin and auxin, and their possible interactions, in nodule development.

## INTRODUCTION

Legumes (Fabaceae) are well-known for their ability to form nodules on their roots through symbiotic interaction with soil bacteria (rhizobia), a relationship termed “root nodule symbiosis.” Within the nodules, the rhizobia fix gaseous nitrogen and make it available to the host plants as a nitrogen source; in turn, the plants provide a carbon source for the rhizobia. Nodule development is a form of cellular reprogramming in which host receptors in the root epidermis respond to rhizobia-derived nodulation (Nod) factors by ultimately inducing the dedifferentiation of some root cortical cells (Szczyglowski et al., 1998; Oldroyd et al., 2011). These activated cortical cells subsequently proliferate to form nodule primordia. Nodule organogenesis proceeds further following the invasion of nodule primordia by rhizobia via specialized structures called infection threads (Murray, 2011). Thus, the analysis of nodulation is not only of interest to researchers studying plant–microbe interactions, but also may contribute to our understanding of mechanisms underlying *de novo* organogenesis in plants.

Elucidation of the roles and functions of phytohormones is crucial to understanding plant development (Durbak et al., 2012). Two of these phytohormones, cytokinin and auxin, are well-known as key players in the regulation of cell proliferation and differentiation processes. In *Arabidopsis thaliana*, the roles of these phytohormones and their crosstalk during lateral root (LR) development have been broadly characterized (Benková and Bielach, 2010; Bishopp et al., 2011). Auxin is involved in the positive regulation of LR development: establishment of a local auxin response at LR founder cells results from polar auxin transport and maintenance of the local auxin maximum at the root apex (Benková et al., 2003; Marhavý et al., 2013). In contrast, cytokinin acts as a negative regulator of LR initiation through promoting the expression of auxin signaling inhibitors (Laplaze et al., 2007; Bielach et al., 2012).

Most of the early studies on the hormonal control of nodulation adopted a physiological approach using a variety of leguminous and rhizobial species. More recently, the advances in genetic techniques have led to a greater focus on model legumes such as *Lotus japonicus* and *Medicago truncatula*. In this review, we summarize past and recent studies, mainly from the latter species, on the actions of cytokinin and auxin in the control of nodule development.

## ROLE OF CYTOKININ DURING NODULE DEVELOPMENT

Forty years ago, Libbenga et al. (1973) reported that exogenous application of cytokinin and auxin to pea root cortical explants induced cell proliferation at positions where nodules were expected to initiate. Other early studies found that some rhizobial species could secrete cytokinin-like compounds affecting plant development in soybean (Phillips and Torrey, 1972; Sturtevant and Taller, 1989). Later, Cooper and Long (1994) reported the important observation that the nodulation-deficient phenotype of a *Rhizobium* mutant could be partially suppressed by the introduction of a gene involved in *trans*-zeatin secretion. In their experiment, they found that nodules formed by alfalfa roots were devoid of bacteria, suggesting that while cytokinin has the ability to form nodules, bacterial infection is not affected by cytokinin. Thus, cytokinin may specifically function in nodule organogenesis and not in the rhizobial infection process. After the identification of the Nod factor as a bona fide regulator of nodulation (Spaink et al., 1991; Truchet et al., 1991), various studies investigated the similarities among cytokinin, rhizobial-inoculation, and Nod factor with respect to their effects on nodulation. With respect to the expression patterns of some early nodulin genes, the identities of the proliferating cortical cells induced by cytokinin appear identical to those induced by rhizobial-inoculation or Nod factor treatment in alfalfa and white clover (Bauer et al., 1996; Fang and Hirsch, 1998; Mathesius et al., 2000). Expression of *EARLY NODULIN 40* (*ENOD40*), the first gene reported to have the ability to induce cortical cell division in *M. truncatula* (Charon et al., 1997), is also activated by cytokinin. These early studies carried out in various legume species reported that nodulation did not progress any further following the stimulation of cortical cell proliferation by cytokinin treatment. Under particular experimental conditions, however, it is possible to stimulate formation of bulges with the appearance of nodule-like primordia by application of cytokinin to roots of *L. japonicus *(Heckmann et al., 2011). Interestingly, the frequency of formation of these structures varies among *Lotus *species, suggesting that there may be an inter-species difference in cytokinin responses.

## IDENTIFICATION OF KEY COMPONENTS OF NODULATION-RELATED CYTOKININ SIGNALING

In *L. japonicus*, mutation at any of three* spontaneous nodule formation* loci (*snf1*, *snf2*, or *snf4*) can cause the formation of nodule-like structures (spontaneous nodules) in the absence of rhizobia (Tirichine et al., 2006b). The histological, physiological, and molecular features of spontaneous nodules resemble those of rhizobia-induced nodules; the major difference is the presence of infection threads and infected cells in the latter. The observation of spontaneous nodules has also been reported in some ecotypes of alfalfa, although the cause remains unknown (Truchet et al., 1989).

The genetic study of cytokinin function during nodule development has been facilitated by use of a mutation at the *snf2 *locus that is associated with spontaneous nodule development. This dominant *snf2* mutant has a gain-of-function mutation of *LOTUS HISTIDINE KINASE 1* (*LHK1*), which encodes a protein closely related to the *Arabidopsis* cytokinin receptor, CYTOKININ RESPONSE 1 (CRE1)/ARABIDOPSIS HISTIDINE KINASE 4 (Inoue et al., 2001; Tirichine et al., 2007). The mutant histidine kinase receptor can activate an *Escherichia coli* two-component phosphorelay system without exogenous cytokinin treatment; this observation suggests that cytokinin-induced signaling is constitutively activated in the *snf2* mutant. *L. japonicus *plants carrying a loss-of-function mutation of *LHK1* and *M*. *truncatula *plants with mutation of* CRE1* (*MtCRE1*), the functional homolog of *LHK1*, are insensitive to cytokinin and show a nodulation-deficient phenotype (Gonzalez-Rizzo et al., 2006; Murray et al., 2007; Plet et al., 2011). These observations strongly indicate that activation of cytokinin signaling is essential for nodule development.

In a downstream part of the cytokinin receptor pathway, a series of two-component phosphorelay systems activate B-type response regulators (RRs), which have a DNA-binding domain and can directly regulate a number of cytokinin primary response genes. Among the cytokinin primary response genes, A-type RRs are believed to act as negative regulators of cytokinin signaling (Heyl and Schmülling, 2003). In *M*. *truncatula*, expression of *MtRR1* (B-type) and *MtRR4* (A-type) is induced by inoculation with rhizobia (Gonzalez-Rizzo et al., 2006). *MtCRE1* and *MtRR4* are expressed at proliferating cortical cells during nodule development, and the upregulation of *MtRR4* expression is dependent on *MtCRE1* (Lohar et al., 2006; Plet et al., 2011), suggesting that *MtRR4* is involved in nodule development in a downstream part of the MtCRE1 signaling pathway. At present, no loss-of-function mutants of nodulation-related RRs have been identified. However, in *M. truncatula*, analyses of the loss- and gain-of-function effects of ETHYLENE RESPONSE FACTOR REQUIRED FOR NODULE DIFFERENTIATION (EFD) showed that it negatively regulates nodulation, potentially through the activation of *MtRR4* (Vernié et al., 2008). This is consistent with the suggestion that MtRR4 acts as a negative regulator of nodule development. In addition, the expression of other A-type RRs can be induced by Nod factor treatment in *M. truncatula* (Op den Camp et al., 2011). Surprisingly, under the experimental conditions used, *MtRR4* was not activated by the Nod factor, suggesting that there might be different downstream responses between rhizobial-inoculation and Nod factor treatment. Furthermore, constitutive activation of *MtRR9*, a newly identified A-type RR, induces cortical cell proliferation, implying that *MtRR9* may have a positive role in the formation of nodules (Op den Camp et al., 2011). MtRR9 function in cytokinin signaling should be, however, clarified by investigation of the effects of loss- and gain-of-function mutations on cytokinin sensitivity.

**FIGURE 1:**
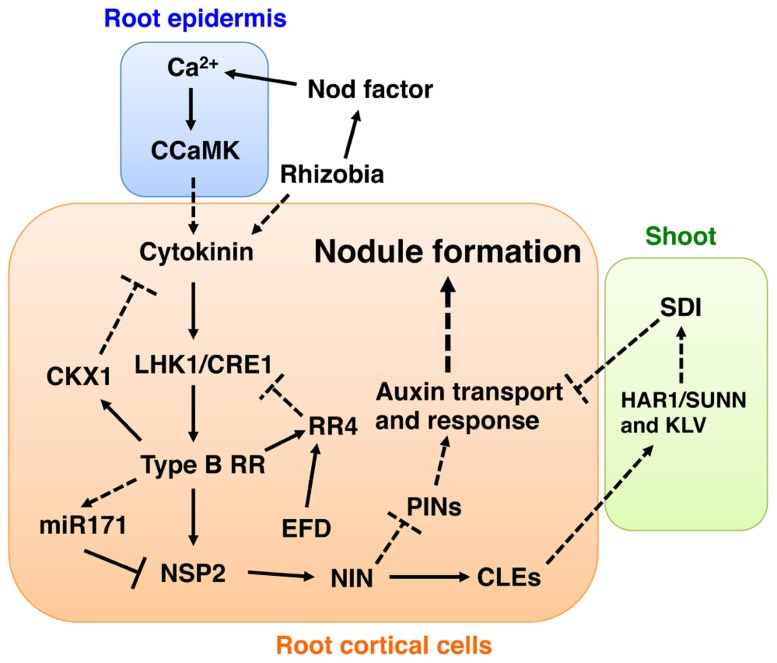
**Model depicting auxin- and cytokinin-mediated signaling pathways involved in nodule development.** To develop this model, we combined data from studies using *L. japonicus* and *M. truncatula*. Based on the observation that *NSP2* is required for the induction of *NIN* (Murakami et al., 2006), we placed NIN downstream of NSP2. Since control of PIN proteins (PINs) localization seems to occur in the downstream part of the cytokinin signaling pathway (Plet et al., 2011) and NIN has an ability to induce localized auxin responses (Suzaki et al., 2012), we suggest that the site of PINs regulation is downstream of NIN. A putative shoot-derived inhibitor (SDI) is proposed as a negative regulatory signal of nodule development mediated by the AON mechanism involving HAR1/SUNN, KLV, and nodulation-related CLE peptides (CLEs) (Ferguson et al., 2010; Kouchi et al., 2010). Here, we suggest that SDI might inhibit cytokinin and auxin actions. Proven and putative regulation points are indicated by intact lines and dotted lines, respectively. See text for more detailed explanations of the model.

Ariel et al. (2012) recently reported that MtRR1 could bind to the *MtRR4* promoter, suggesting that MtRR1 directly controls the expression of *MtRR4*. Interestingly, electrophoretic mobility shift and chromatin immunoprecipitation assays identified* NODULATION SIGNALING PATHWAY 2* (*NSP2*) as a direct target of MtRR1. *NSP2* encodes a GRAS-type transcription factor that is required for the positive regulation of nodule development (Kaló et al., 2005; Heckmann et al., 2006; Murakami et al., 2006; Ariel et al., 2012). Mutation of the putative MtRR1-binding sites of the *NSP2* promoter abolished nodulation-related activation of *NSP2, *suggesting that these *cis*-elements are essential for *NSP2* expression. The regulatory mechanism for *NSP2 *expression is currently a vibrant area of research in plant–microbe interactions; recent evidence indicates that expression of *NSP2 *is negatively regulated by microRNA 171 (miR171; De Luis et al., 2012; Lauressergues et al., 2012). Expression of *miR171* is induced not only during nodule development but also by cytokinin in an *MtCRE1*-dependent manner, and the expression pattern is negatively correlated with that of *NSP2* (Ariel et al., 2012). Thus, cytokinin signaling may have a dual mode for regulating *NSP2 *expression: it can directly activate *NSP2 *transiently and then repress its expression through activation of *miR171* expression (**Figure 1**). Ariel et al. (2012) found that MtRR1 additionally appears to directly regulate a basic helix-loop-helix transcription factor (bHLH476), and that insertion of a *Tnt1 *retrotransposon into *bHLH476 *led to reduced nodulation. This observation suggests that bHLH476 positively regulates nodulation. Another candidate MtRR1 target is *M*. *truncatula* *CYTOKININ OXIDASE 1 *(*MtCKX1*), which is involved in negative regulation of cytokinin signaling (Ariel et al., 2012). MtRR1 binds directly to the *MtCKX1 *promoter in an *MtCRE1-*dependent manner. *CKX* genes have a negative effect on**nodule development and their overexpression causes a reduction in the number of nodules (Lohar et al., 2004). Overall, these findings indicate that cytokinin signaling not only positively regulates nodule development but also may control itself through a negative feedback mechanism that may involve *CKX1 *(**Figure 1**).

Double mutant analyses using *snf2* and nodulation-deficient mutants indicate that *NODULE INCEPTION* (*NIN*) is also involved in the positive regulation of nodule development in a downstream part of the *LHK1*-dependent cytokinin signaling pathway. The *nin* mutation suppresses *snf2*-dependent spontaneous nodule formation (Tirichine et al., 2007). Expression of *NIN* is strongly activated during nodulation and is induced by cytokinin in an *LHK1*/*MtCRE1*-dependent manner (Schauser et al., 1999; Murray et al., 2007; Heckmann et al., 2011; Plet et al., 2011). In addition, *NIN* has the ability to induce cortical cell proliferation in *L. japonicus*; constitutive activation of the gene**induces cortical cell proliferation in the absence of rhizobia (Suzaki et al., 2012; Soyano et al., 2013). Details of the mechanism of the potential interaction between cytokinin signaling and *NIN* activation await clarification.

## RELATIONSHIP BETWEEN CYTOKININ SIGNALING AND AUTOREGULATION OF NODULATION

It has been demonstrated that legumes have a negative regulatory mechanism termed autoregulation of nodulation (AON) that moderates the number of nodules (Caetano-Anollés and Gresshoff, 1991; Oka-Kira and Kawaguchi, 2006; Ferguson et al., 2010; Kouchi et al., 2010). In *L. japonicus* and *M. truncatula*, a key component of AON is long-distance communication between the root and shoot that is mediated through the receptor-like kinases HYPERNODULATION ABERRANT ROOT FORMATION 1 (HAR1)/SUPER NUMERIC NODULES (SUNN) and KLAVIER (KLV) in the shoot and the potential root-derived signals *L. japonicus* CLE-ROOT SIGNAL 1/2 (LjCLE-RS1/2) or MtCLE12/13 (Krusell et al., 2002; Nishimura et al., 2002; Schnabel et al., 2005; Okamoto et al., 2009; Miyazawa et al., 2010; Mortier et al., 2010). Mutation of *HAR1* or *KLV *causes a hypernodulation phenotype in* L. japonicus*; moreover, these mutations have an additive effect on *snf2*-dependent spontaneous nodule formation (Wopereis et al., 2000; Tirichine et al., 2007; Miyazawa et al., 2010), suggesting that AON acts in parallel to the cytokinin signaling pathway that includes LHK1. The expression of nodulation-related *CLE *genes is induced upon rhizobial-inoculation (Okamoto et al., 2009; Mortier et al., 2010), and it has recently been shown that such activation is abolished in the presence of *cre1 *and *nin *mutations in *M.* *truncatula *(Mortier et al., 2012). Thus, the CLE peptides may be produced in the downstream part of the cytokinin signaling pathway that involves *NIN*. Several studies have demonstrated that nodulation is strongly suppressed when the *CLE *genes are constitutively activated (Okamoto et al., 2009; Mortier et al., 2010, 2012). In order to further understand the potential feedback regulation between cytokinin signaling and AON, it will be necessary to determine the effects of *CLE* expression on *snf2*-dependent spontaneous nodulation.

## RELATIONSHIP BETWEEN AUXIN AND GENETIC PATHWAYS THAT CONTROL NODULE DEVELOPMENT

Allen et al. (1953) were the first to show that exogenous application of polar auxin transport inhibitors to alfalfa roots induced formation of nodule-like structures in the absence of rhizobia. Subsequent investigations on the expression of early nodulin genes and of their expression profiles during pseudonodules development suggest that they are similar to rhizobia-induced nodules in the genus *Medicago* (Hirsch et al., 1989; Hirsch and Fang, 1994; Rightmyer and Long, 2011). An auxin reporter analysis using the *GH3* promoter showed that the Nod factor is able to perturb auxin flow in white clover (Mathesius et al., 1998). Furthermore, deficiency in flavonoids, which act to inhibit auxin transport, causes a reduction in nodule number in *M. truncatula* (Wasson et al., 2006). Overall, these observations suggest that alteration of the auxin flow affects nodule development, thereby implicating auxin in this process.

Recently, the highly active synthetic auxin-responsive element* DR5* has been used in combination with a nuclear-localized green fluorescent protein (GFP) as a reporter to examine auxin response patterns during *L. japonicus* nodule development (Suzaki et al., 2012). The analysis revealed that auxin responses during nodule development exclusively occur in proliferating cortical cells, as also reported by previous studies using the *GH3 *promoter**(Pacios-Bras et al., 2003; Takanashi et al., 2011). An auxin response was also observed in *cyclops* mutants, in which infection threads fail to reach cortical cells (Yano et al., 2008). Thus, formation of infection threads may not be required for initiation of the auxin response. During actinorhizal nodule formation in *Casuarina glauca*, the localized accumulation of auxin is mediated by AUX1-like carriers and is correlated with the cellular infection by bacteria (Péret et al., 2007; Perrine-Walker et al., 2010). Localized auxin responses are induced during *snf2*-dependent spontaneous nodule formation, suggesting that cytokinin signaling has a role in the production of these responses (Suzaki et al., 2012). A localized auxin response is also observed during spontaneous nodule development mediated by a gain-of-function mutation of the Ca^2^^+^/calmodulin-dependent protein kinase (CCaMK; Suzaki et al., 2013), which is responsible for decoding Ca^2^^+^ signals during nodulation (Gleason et al., 2006; Tirichine et al., 2006a; Hayashi et al., 2010; Madsen et al., 2010; Shimoda et al., 2012). Since accumulation of MtPIN proteins, coding putative auxin efflux carriers, appears to be negatively regulated by *MtCRE1*-dependent cytokinin signaling**(Plet et al., 2011), the regulation of the polar localization of some PIN proteins may be required for the establishment of localized auxin responses. In *A. thaliana*, cytokinin inhibits the initiation of LR development by blocking the expression of *PIN *genes in LR founder cells (Laplaze et al., 2007). Thus, the negative regulation of PIN auxin carriers by cytokinin might be conserved in nodule and LR development. In legumes, cytokinin promotes nodule development (as described above) but also inhibits LR formation (Lohar et al., 2004; Gonzalez-Rizzo et al., 2006). During formation of spontaneous structures induced by constitutive activation of *NIN*, localized auxin responses are also induced in *L. japonicus* (Suzaki et al., 2012). Thus, it is highly likely that localized auxin responses occur not only downstream of CCaMK and LHK1 but also of NIN (**Figure 1**). This interpretation is consistent with the observation that the *nin* mutation has no effect on pseudonodule formation induced by auxin transport inhibitors (Rightmyer and Long, 2011).

Recently, a *DR5* reporter analysis in *har1 *mutants of *L. japonicus* indicated that *HAR1 *may negatively regulate auxin responses during nodule development (Suzaki et al., 2012). Abnormal auxin transport may underlie the higher auxin response in *har1* mutants as *sunn *mutants have an increased auxin transport from the shoot to the root (van Noorden et al., 2006). In addition, in nodulation-deficient roots resulting from the constitutive activation of *LjCLE* genes, perturbation of cortical cell proliferation is accompanied by the disappearance of auxin responses (Suzaki et al., 2012). Thus, it is possible that AON may negatively regulate nodule development through controlling auxin responses (**Figure 1**).

## FUTURE PERSPECTIVES

As we show in this mini-review, significant progress has been made recently in our understanding of how and when cytokinin and auxin act in the various genetic pathways that control nodule development. Although auxin has a longer history than cytokinin with respect to research into root nodule symbiosis, there is comparatively little known of its role in nodule development due to a dearth of auxin-related mutants involved in nodulation. In *M.* *truncatula*, however, the characterization of the *Mtpin1* (*smooth leaf margin 1*) nodulation-phenotype may help remedy this situation (Zhou et al., 2011). Additionally, characterization of mutants created by retrotransposon mutagenesis (*LORE1* in *L. japonicus* and *Tnt1* in *M.* *truncatula*; Fukai et al., 2012; Pislariu et al., 2012; Urbański et al., 2012) should accelerate genetic studies of nodule development. In the current model of nodule development, it is proposed that auxin accumulates in the incipient nodule primordia under the control of auxin transport (**Figure 1**). However, we cannot rule out the possibility of *de novo* auxin production as expression of a putative auxin biosynthesis gene is activated during nodule development in *L. japonicus* (Suzaki et al., 2012). With regard to cytokinin, a recent study has shown that activation of some genes involved in cytokinin biosynthesis, degradation, and conjugation is correlated with nodule development in *M.* *truncatula* (Moreau et al., 2011). In addition to studies of plant phytohormones, it is possible that investigation of auxin- and cytokinin-like compounds derived from the rhizobia may provide new insights into nodule development. Some species of rhizobia do not possess genes to synthesize Nod factors but instead might use cytokinin-like compounds to establish root nodule symbiosis (Giraud et al., 2007). In order to elucidate how cytokinin and auxin are provided during nodule development, it will be necessary to investigate the functions of host and rhizobial genes involved in the production of these phytohormones.

## Conflict of Interest Statement

The authors declare that the research was conducted in the absence of any commercial or financial relationships that could be construed as a potential conflict of interest.
